# Prediction of mechanical complications post-acute myocardial infarction in individuals with type 2 diabetes mellitus

**DOI:** 10.3389/fmed.2025.1635357

**Published:** 2025-08-01

**Authors:** Changying Zhao, Han Wang, Wei Yuan, Yang Yan

**Affiliations:** ^1^Department of Cardiovascular Surgery, The First Affiliated Hospital of Xi’an Jiaotong University, Xi’an, China; ^2^Xi’an Jiaotong University Health Science Center, Xi’an, China; ^3^Department of Cardiovascular Medicine, The First Affiliated Hospital of Xi’an Jiaotong University, Xi’an, China

**Keywords:** acute myocardial infarction, type 2 diabetes mellitus, mechanical complications, prediction model, hemoglobin A1c

## Abstract

**Background:**

Acute myocardial infarction (AMI) patients with type 2 diabetes mellitus (T2DM) represent a unique population characterized by poorer prognoses, which may be further exacerbated by mechanical complications. This study aims to develop a predictive model to identify high-risk individuals within this populations.

**Methods:**

This study enrolled AMI patients with T2DM and categorized them into complication and control groups. The mechanical complications were defined as papillary muscle rupture (with or without acute mitral regurgitation), ventricular septal defect, left ventricular pseudoaneurysm or aneurysm (with or without thrombus) and free wall rupture. Characteristics were selected using relaxed least absolute shrinkage and selection operator (LASSO) logistic regression, multivariate logistic regression and random forest model. Selected variables were utilized to construct a nomogram to predict the possibility of mechanical complications.

**Results:**

A total of 2,816 patients were enrolled, with 191 individuals classified into the complication group. Baseline analysis identified 31 factors exhibiting potential differences, which were subsequently employed for LASSO-logistic regression, multivariate logistic regression and random forest model. After comprehensive evaluation, nine variables emerged as predictive factors for mechanical complications, including gender, pulmonary hypertension, ST-segment elevation myocardial infarction, body mass index, N-terminal pro-brain natriuretic peptide, creatine kinase, left ventricle ejection fraction and hemoglobin A1c, which were used to construct a reliable nomogram. The complication group also showed higher in-hospital mortality rates compared to controls, alerting the worse prognosis of these populations.

**Conclusion:**

This study identified nine factors upon admission that may be associated with mechanical complications during the hospitalization. A nomogram was developed based on these factors for clinical application. T2DM patients should emphasize glucose control, which may offer benefits following the onset of AMI.

## 1 Introduction

Acute myocardial infarction (AMI) is a disease characterized by the rupture of atherosclerotic plaques, resulting in coronary artery occlusion and subsequent myocardial ischemia and infarction (1). Currently, direct percutaneous coronary intervention has emerged as the standard treatment for AMI, effectively reducing infarct size and significantly improving early survival rates among AMI patients (2). Type 2 diabetes mellitus (T2DM) is recognized as a risk factor for AMI (3). The incidence of cardiovascular diseases in adults with T2DM is two to three times higher than that in their non-diabetic counterparts. Furthermore, studies indicate that the mortality rate within 5 years post-AMI for T2DM patients is twice that of other individuals, potentially reaching as high as 50% (4). Consequently, AMI patients with a history of T2DM represent a distinct population at elevated risk, necessitating heightened attention to enhance prognosis.

The overall incidence of mechanical complications following AMI ranges from approximately 0.1 to 0.3%, mainly including papillary muscle rupture (with or without acute mitral regurgitation), ventricular septal defect, pseudoaneurysm, among others (5, 6). Despite advancements in reperfusion therapies, continue to serve as critical determinants of clinical results in AMI patients (7, 8). Researches indicated that the risks associated with in-hospital death, cardiogenic shock, acute kidney injury and respiratory complications are all markedly higher among patients who experience mechanical complications compared to those who do not (5, 9). Identifying high-risk AMI patients is essential for the early prevention and management of these mechanical complications.

Given the lack of literature addressing the prediction of mechanical complications, this study aims to construct a model to predict the possibility of mechanical complications during the hospitalization in AMI patients with T2DM at admission. This study may assist clinicians in distinguishing high-risk individuals more effectively, helping hierarchical management of patients and early interventions.

## 2 Methods

### 2.1 Patient enrollment

Patients who were hospitalized at the First Affiliated Hospital of Xi’an Jiaotong University from January 2020 to December 2024 due to AMI combined with T2DM were included in this study. The diagnosis of AMI was established according to the guidelines set forth by European Society of Cardiology. The exclusion criteria mainly consisted of: (1) patients with a history of severe comorbidities that may affect the occurrence of the outcomes, such as hepatic dysfunction; (2) other subtypes of diabetes or unclassifiable diabetes (e.g., type 1 diabetes mellitus, gestational diabetes mellitus); (3) patients with incomplete clinical data that could not be addressed through statistical methods.

This study was approved by the Ethics Committee of the First Affiliated Hospital of Xi’an Jiaotong University (XJTU1AF2025LSYY-505; date: 9 May 2025), in accordance with the Declaration of Helsinki. The Ethics Committee of the First Affiliated Hospital of Xi’an Jiaotong University granted a waiver for informed consent to this study due to its retrospective design.

### 2.2 Data collection and grouping

The basic demographic information, biochemical test results, imaging data and in-hospital outcomes were obtained from the Biobank of the First Affiliated Hospital of Xi’an Jiaotong University. Enrolled patients were categorized into complication and control groups based on the occurrence of in-hospital mechanical complications. To enhance the selection of high-risk patients, the definition of mechanical complications was broadened to encompass papillary muscle rupture (with or without acute mitral regurgitation), ventricular septal defect, left ventricular pseudoaneurysm or aneurysm (with or without thrombus) and free wall rupture. These definitions included both traditional acute complications and chronic conditions associated with mechanical complications (5, 10).

### 2.3 Statistical analysis

Baseline variables with more than 20% missing values were excluded from further analysis, such as platelet counts (30.0%), left ventricular end-diastolic and end-systolic diameter (44.5% and 44.5%), and cardiac output (53.8%). The remaining variables were missing at random and were addressed using multiple interpolation by chained equations techniques with 5 iterations. A total of five imputed datasets generated, and the fifth dataset without missing values was used for following analysis. Continuous variables, following normal distribution, were presented as mean ± standard deviation and compared using Student’s t-tests. Other continuous variables were represented as median with interquartile range and were analyzed using Mann–Whitney U tests. Categorical variables were reported as absolute counts with relative frequencies. Chi-square tests and Fisher’s exact tests were employed for comparisons. The relaxed least absolute shrinkage and selection operator (LASSO) logistic regression model was used for preliminary screening of variables which had a *p* value less than 0.10 in the baseline characteristics. Screened variables underwent further examination through multivariate logistic regression and random forest model. Random forest modeling included 500 trees, with variable importance ranked by the arithmetic means of the accuracy and Gini index values. Factors demonstrating significant differences in either the multivariate or random forest models were included to construct a nomogram. The receiver operating characteristic curve, calibration curve and decision curve were employed to evaluate the performance of nomogram. Data analysis was conducted using SPSS software (version 27.0) and R software (version 4.1.1). *P*-value less than 0.05 was identified as having statistical difference.

## 3 Results

### 3.1 Baseline characteristics and clinical outcomes

**Figure 1:**
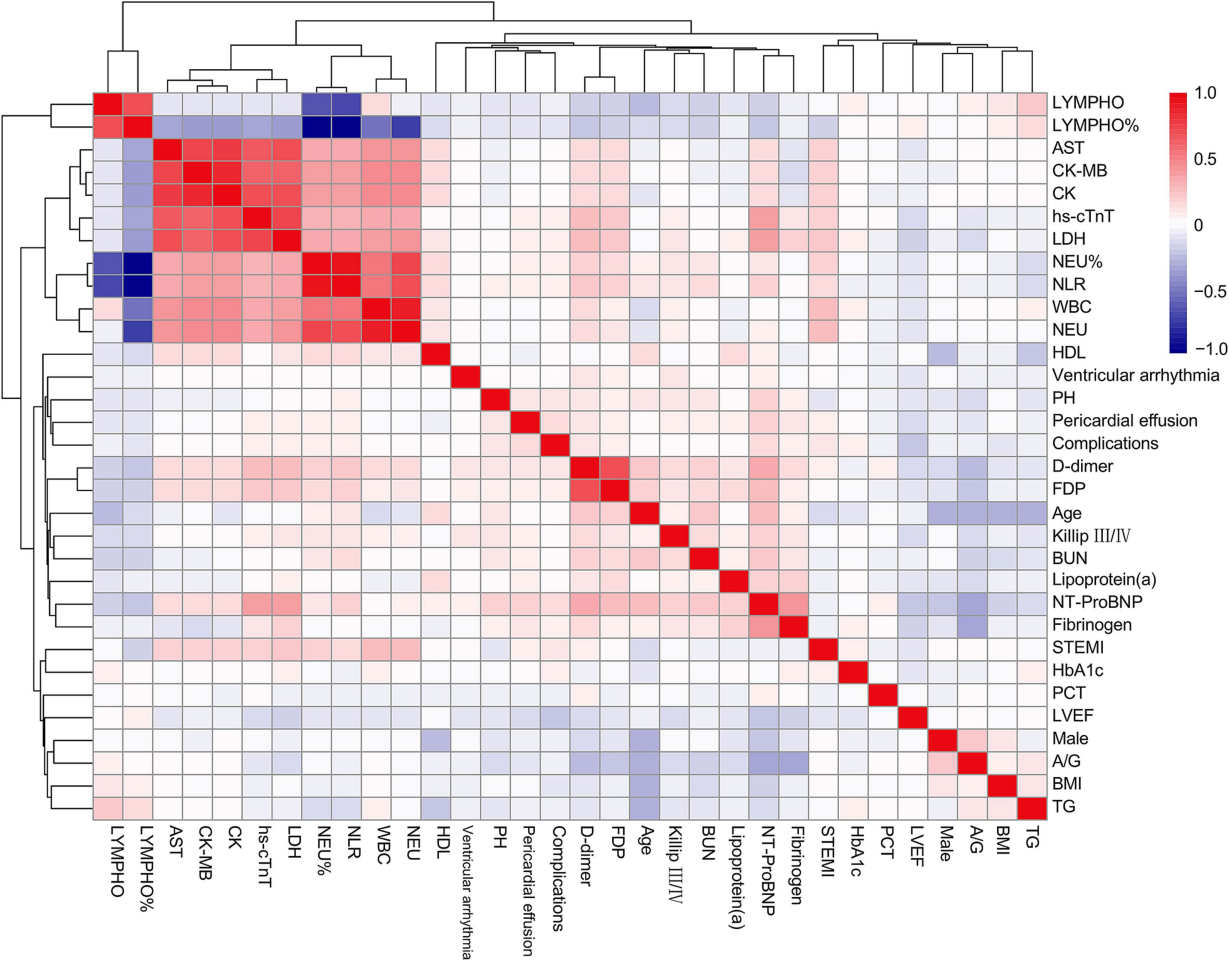
Correlation heatmap between selected variables and the incidence of complications. LYMPH, lymphocyte count; AST, aspartate aminotransferase; CK-MB, creatine kinase isoenzymes MB; CK, creatine kinase; hs-cTnT, high-sensitivity cardiac troponin T; LDH, lactate dehydrogenase; NEU, neutrophil count; NLR, neutrophil-to-lymphocyte ratio; WBC, white blood cell; HDL, high-density lipoprotein; PH, pulmonary hypertension; FDP, fibrin degradation products; BUN, blood urea nitrogen; NT-ProBNP, N-terminal pro-brain natriuretic peptide; STEMI, ST-segment elevation myocardial infarction; HbA1c, hemoglobin A1c; PCT, procalcitonin; LVEF, left ventricle ejection fraction; A/G, albumin/globulin ratio; BMI, body mass index; TG, triglycerides.

A total of 2,816 patients with AMI complicated by T2DM were enrolled in this study, among whom 191 (6.78%) developed in-hospital mechanical complications. The baseline characteristics of both groups were summarized in Table 1. In comparison to the control group, the complication group exhibited higher proportions of female gender, ST-segment elevation myocardial infarction (STEMI), and Killip class III/IV classification. Additionally, this group demonstrated elevated levels of age, body mass index (BMI), hemoglobin A1c (HbA1c), neutrophil count, lymphocyte count, neutrophil-to-lymphocyte ratio, aminotransferase, globulin, albumin/globulin ratio, lipoprotein (a), N-terminal pro-brain natriuretic peptide (NT-ProBNP), high-sensitivity cardiac troponin T, lactate dehydrogenase, fibrinogen, D-dimer, and fibrin degradation products. Conversely, this group displayed lower levels of left ventricular ejection fraction (LVEF) and triglycerides when compared to the control group. Furthermore, correlation analysis of the incidence of mechanical complications and parameters which might relate to mechanical complications (*p* < 0.10) in baseline was illustrated in Figure 1.

**Table 1:** Baseline characteristics and in-hospital clinical outcomes between the two groups.

Items	Total	Complication group	Control group	*p* value
	(*n* = 2,816)	(*n* = 191)	(*n* = 2,625)	
Age (years)	62.50 (54.00, 70.00)	64.00 (55.00, 71.00)	62.00 (54.00, 70.00)	0.043
Gender (male, %)	2,141 (76.0)	129 (67.5)	2012 (76.6)	0.004
BMI (kg/m^2^)	24.74 (22.57, 26.89)	23.94 (21.38, 26.12)	24.48 (22.65, 26.91)	0.001
Body temperature (°C)	36.30 (36.00, 36.50)	36.30 (36.00, 36.50)	36.30 (36.00, 36.50)	0.887
Smoking (%)	887 (31.5)	56 (29.3)	831 (31.7)	0.502
Drinking (%)	408 (14.5)	21 (11.0)	387 (14.7)	0.155
STEMI (%)	1,557 (55.3)	140 (73.3)	1,417 (54.0)	<0.001
Killip III/IV (%)	171 (6.1)	20 (10.5)	151 (5.8)	0.008
Comorbidities (%)				
Hypertension	976 (34.7)	62 (32.5)	914 (34.8)	0.508
Diabetic complications	164 (5.8)	13 (6.8)	151 (5.8)	0.548
Hyperlipidemia	228 (8.1)	12 (6.3)	216 (8.2)	0.341
CKD	121 (4.3)	6 (3.1)	115 (4.4)	0.415
Biochemical results				
Hb (g/L)	139.00 (125.00, 151.00)	137.00 (124.50, 151.00)	139.00 (125.00, 151.00)	0.555
HbA1c (%)	7.80 (6.90, 9.20)	8.20 (7.10, 9.70)	7.80 (6.90, 9.20)	0.003
WBC (10^9^/L)	8.72 (6.82, 11.08)	9.10 (6.84, 12.15)	8.69 (6.82, 11.00)	0.099
NEU (10^9^/L)	6.39 (4.67, 8.80)	6.95 (4.90, 10.09)	6.34 (4.65, 8.74)	0.012
NEU% (%)	75.00 (67.90, 82.10)	77.40 (70.63, 83.80)	74.90 (67.5, 81.99)	0.001
LYMPH (10^9^/L)	1.46 (1.08, 1.92)	1.35 (1.00, 1.69)	1.47 (1.08, 1.94)	0.005
LYMPH% (%)	17.67 (11.97, 23.82)	15.11 (10.06, 21.54)	17.76 (12.13, 23.98)	<0.001
NLR	4.28 (2.83, 6.89)	5.24 (3.32, 8.40)	4.21 (2.81, 6.76)	<0.001
CRP (mg/L)	61.14 (31.09, 97.15)	62.75 (31.80, 100.82)	61.10 (30.97, 97.00)	0.851
hs-CRP (mg/L)	2.42 (1.13,4.70)	2.31 (1.21, 3.82)	2.42 (1.13, 4.72)	0.976
PCT (ng/mL)	1.44 (0.11, 3.85)	0.88 (0.10, 3.36)	1.48 (0.11, 3.92)	0.074
ALT (U/L)	30.00 (20.00, 46.00)	30.00 (20.00,46.00)	32.00 (21.00, 48.00)	0.224
AST (U/L)	41.00 (24.00,93.00)	50.00 (25.00, 127.00)	40.29 (24.00, 91.00)	0.015
Total protein (g/L)	63.70 (59.10, 68.20)	63.10 (57.20, 69.40)	63.70 (59.20, 68.10)	0.322
Albumin (g/L)	37.70 (34.50, 41.10)	37.10 (32.15, 41.48)	37.80 (34.60, 41.10)	0.570
Globulin (g/L)	25.80 (23.40, 28.50)	26.20 (23.90, 29.10)	25.80 (23.33, 28.50)	0.136
A/G	1.46 (1.29, 1.64)	1.41 (1.21, 1.61)	1.46 (1.29, 1.65)	0.019
eGFR (mL/min/1.73m^2^)	94.25 (72.81, 104.61)	91.94 (65.11,103.61)	94.51 (73.32, 104.65)	0.149
Cr (umol/L)	65.00 (53.00, 82.00)	65.00 (53.00, 84.75)	65.00 (53.00, 82.00)	0.764
BUN (mmol/L)	6.07 (4.80, 7.74)	6.32 (5.07, 8.01)	6.06 (4.79, 7.72)	0.085
LDL (mmol/L)	2.25 (1.70, 2.88)	2.25 (1.67, 2.92)	2.25 (1.70, 2.87)	0.876
HDL (mmol/L)	0.88 (0.76, 1.02)	0.94 (0.75, 1.08)	0.88 (0.76, 1.02)	0.034
TG (mmol/L)	1.43 (1.01, 2.11)	1.29 (0.95, 1.86)	1.44 (1.02, 2.13)	0.011
TC (mmol/L)	3.96 (3.26, 4.81)	4.05 (3.20, 4.85)	3.96 (3.27, 4.81)	0.931
Lipoprotein (a) (mg/L)	220.50 (118.25, 414.26)	277.57 (140.00, 488.00)	217.00 (117.79, 412.00)	0.005
ApoE (mg/L)	34.40 (27.10, 44.55)	34.60 (27.20, 44.00)	34.40 (27.08, 44.63)	0.928
ApoB (g/L)	0.80 (0.65, 0.97)	0.81 (0.66, 0.98)	0.80 (0.65, 0.97)	0.698
ApoA (g/L)	1.03 (0.92, 1.16)	1.04 (0.91, 1.18)	1.03 (0.92, 1.15)	0.530
NT-ProBNP (pg/mL)	930.00 (295.13, 2709.00)	2452.00 (873.50, 4888.00)	870.00 (279.00, 2555.51)	<0.001
hs-cTnT (ng/mL)	0.49 (0.11, 1.62)	1.03 (0.31, 2.29)	0.47 (0.11, 1.53)	<0.001
CK-MB (U/L)	26.20 (15.00, 77.00)	15.00 (11.30, 29.80)	15.00 (10.00, 26.00)	0.080
CK (U/L)	232.00 (93.25, 722.95)	95.00 (62.00, 299.00)	93.00 (60.00, 227.00)	0.076
LDH (U/L)	277.50 (219.00, 419.71)	346.00 (239.00, 545.00)	276.00 (217.00, 408.00)	<0.001
APTT (s)	31.40 (26.70, 37.20)	29.92 (26.20, 36.57)	31.40 (26.70, 37.30)	0.141
PT (s)	13.10 (12.10, 13.90)	13.10 (12.05, 14.00)	13.10 (12.10, 13.80)	0.924
PTA (%)	97.00 (85.20, 108.00)	95.00 (84.00, 108.00)	97.00 (85.50, 108.00)	0.575
INR	1.02 (0.97, 1.09)	1.03 (0.98, 1.10)	1.02 (0.97, 1.08)	0.152
APTR	1.02 (0.93, 1.15)	1.00 (0.93, 1.12)	1.02 (0.93, 1.16)	0.158
Fibrinogen (g/L)	3.55 (2.87, 4.52)	3.81 (3.01, 4.99)	3.53 (2.86, 4.47)	0.001
D-dimer (mg/L)	0.56 (0.31, 1.10)	0.80 (0.44, 1.80)	0.54 (0.30, 1.07)	<0.001
FDP (mg/L)	1.99 (1.22, 4.60)	2.79 (1.54, 9.49)	1.93 (1.20, 4.31)	<0.001
Echocardiology				
LVEF (%)	52.00 (44.00, 60.00)	43.50 (39.70, 48.00)	52.50 (45.00, 60.00)	<0.001
PH (%)	121 (4.3)	24 (12.6)	97 (3.7)	<0.001
Pericardial effusion (%)	181 (6.4)	37 (19.4)	144 (5.5)	<0.001
ECG (%)				
Ventricular arrhythmia	119 (4.2)	13 (6.8)	106 (4.0)	0.066
Atrioventricular block	118 (4.2)	5 (2.6)	113 (4.3)	0.261
In-hospital outcomes				
IABP (%)	124 (4.4)	23 (12.0)	101 (3.8)	<0.001
ECMO (%)	32 (1.1)	6 (3.1)	26 (1.0)	0.007
PCI (%)	2,422 (86.0)	161 (84.3)	2,261 (86.1)	0.479
CABG (%)	24 (0.9)	2 (1.0)	22 (0.8)	0.762
Temporary pacemaker (%)	42 (1.5)	2 (1.0)	40 (1.5)	0.600
CRRT (%)	57 (2.0)	5 (2.6)	52 (2.0)	0.546
Length of hospital stay (days)	5.00 (3.00, 7.00)	5.00 (3.00, 8.00)	5.00 (3.00, 7.00)	0.159
In-hospital mortality (%)	31 (1.1)	5 (2.6)	26 (1.0)	0.037

BMI, body mass index; STEMI, ST-segment elevation myocardial infarction; CKD, chronic kidney disease; Hb, hemoglobin; HbA1c, hemoglobin A1c; WBC, white blood cell; NEU, neutrophil count; LYMPH, lymphocyte count; NLR, neutrophil-to-lymphocyte ratio; CRP, C-reactive protein; hs-CRP, high-sensitivity C-reactive protein; PCT, procalcitonin; ALT: alanine aminotransferase; AST, aspartate aminotransferase; A/G, albumin/globulin ratio; eGFR, estimated glomerular filtration rate; Cr, creatinine; BUN, blood urea nitrogen; LDL, low-density lipoprotein; HDL, high-density lipoprotein; TG, triglycerides; TC, total cholesterol; ApoE, apolipoprotein E; ApoB, apolipoprotein B; ApoA, apolipoprotein A; NT-ProBNP, N-terminal pro-brain natriuretic peptide; hs-cTnT, high-sensitivity cardiac troponin T; CK-MB, creatine kinase isoenzymes MB; CK, creatine kinase; LDH, lactate dehydrogenase; APTT, activated partial thromboplastin time; PT, prothrombin time; PTA, prothrombin activity; INR, international normalized ratio; APTR, activated partial thromboplastin ratio; FDP, fibrin degradation products; LVEF, left ventricle ejection fraction; PH, pulmonary hypertension; ECG, electrocardiogram; IABP, intra-aortic balloon pump; ECMO, extracorporeal membrane oxygenation; PCI, percutaneous coronary intervention; CABG, coronary artery bypass grafting; CRRT, continuous renal replacement therapy.

Additionally, the complication group exhibited a higher proportion of intra-aortic balloon pump [23 (12.0) vs. 101 (3.8), *p* < 0.001] and extracorporeal membrane oxygenation [6 (3.1) vs. 26 (1.0), *p* = 0.007] utilization, along with an elevated levels of in-hospital mortality [5 (2.6) vs. 26 (1.0), *p* = 0.037] (Table 1). Cardiac rupture was confirmed or suspected in all fatal cases in complication group. One patient developed ventricular septal rupture complicated by ventricular aneurysm and was supported with intra-aortic balloon pump. During the discussion regarding surgical intervention of the family, the patient experienced sudden ventricular fibrillation followed by cardiogenic shock and eventually died. Another patient presented with ventricular aneurysm and developed acute cardiogenic shock. Despite combined support with extracorporeal membrane oxygenation and intra-aortic balloon pump, the patient did not survive. Three additional patients were diagnosed with ventricular aneurysm, one of whom received extracorporeal membrane oxygenation support. However, all three patients suddenly lost consciousness during hospitalization, with concomitant hypotension. Echocardiography revealed large pericardial effusion, and cardiac rupture was suspected as the cause of death.

### 3.2 LASSO-logistic regression results

**Figure 2:**
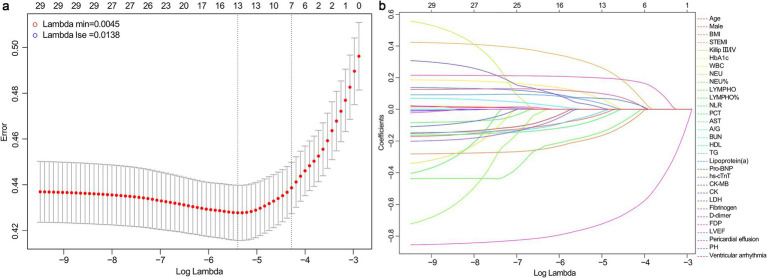
Results of LASSO-logistic regression. **(a)** Cross-validation plot of LASSO-logistic regression. **(b)** Selection process of LASSO-logistic regression model by cross-validation method. BMI, body mass index; STEMI, ST-segment elevation myocardial infarction; HbA1c, hemoglobin A1c; WBC, white blood cell; NEU, neutrophil count; LYMPH, lymphocyte count; NLR, neutrophil-to-lymphocyte ratio; PCT, procalcitonin; AST, aspartate aminotransferase; A/G, albumin/globulin ratio; BUN, blood urea nitrogen; HDL, high-density lipoprotein; TG, triglycerides; NT-ProBNP, N-terminal pro-brain natriuretic peptide; hs-cTnT, high-sensitivity cardiac troponin T; CK-MB, creatine kinase isoenzymes MB; CK, creatine kinase; LDH, lactate dehydrogenase; FDP, fibrin degradation products; LVEF, left ventricle ejection fraction; PH, pulmonary hypertension.

Considering the correlation among potential variables, characteristics demonstrating statistical differences were incorporated into the LASSO-logistic regression analysis. When lambda.min = 0.0045 and log (lambda.min) = −5.3468, 13 variables were preliminary selected for further evaluation (Figure 2; Table 2).

**Table 2:** Results pf LASSO-logistic regression model.

Items	LASSO-logistic regression	
	Assignment	Coefficient
Age (years)	Continuous variable	
Gender (male, %)	Male = 1, Female = 0	−0.1245
BMI (kg/m^2^)	Continuous variable	−0.2571
STEMI (%)	Yes = 1, No = 0	0.3920
Killip III/IV (%)	Yes = 1, No = 0	
HbA1c (%)	Continuous variable	0.1797
WBC (10^9/L)	Continuous variable	
NEUT (10^9/L)	Continuous variable	
NEUT% (%)	Continuous variable	
LYMPH (10^9/L)	Continuous variable	−0.2476
LYMPH% (%)	Continuous variable	
NLR	Continuous variable	
PCT (ng/ml)	Continuous variable	−0.1671
AST (U/L)	Continuous variable	
Globulin (g/L)	Continuous variable	
A/G	Continuous variable	
BUN (mmol/L)	Continuous variable	
HDL (mmol/L)	Continuous variable	
TG (mmol/L)	Continuous variable	−0.1389
Lipoprotein (a) (mg/L)	Continuous variable	0.1096
NT-ProBNP (pg/mL)	Continuous variable	0.0800
hs-cTnT (ng/mL)	Continuous variable	
CK-MB (U/L)	Continuous variable	
CK (U/L)	Continuous variable	0.0825
LDH (U/L)	Continuous variable	
Fibrinogen (g/L)	Continuous variable	
D-dimer (mg/L)	Continuous variable	
FDP (mg/L)	Continuous variable	
LVEF (%)	Continuous variable	−0.8338
PH (%)	Yes = 1, No = 0	0.1335
Pericardial effusion (%)	Yes = 1, No = 0	0.2133
Ventricular arrhythmia (%)	Yes = 1, No = 0	

LASSO, least absolute shrinkage and selection operator; other abbreviations as in Table 1.

### 3.3 Multivariate logistic regression and random forest model

After the LASSO-logistic regression analysis, multivariate logistic regression analysis was conducted (Table 3). The results indicated that male gender (OR = 0.66, 95%CI: 0.47–0.94; *p* = 0.020); BMI (OR = 0.93, 95%CI: 0.89–0.98; *p* = 0.004); STEMI (OR = 2.23, 95%CI: 1.55–3.20; *p* < 0.001); HbA1c (OR = 1.11, 95%CI: 1.03–1.21; *p* = 0.010); LVEF (OR = 0.92, 95%CI: 0.91–0.94; *p* < 0.001); pulmonary hypertension (PH) (OR = 2.36, 95%CI: 1.36–4.09; *p* = 0.002); pericardial effusion (OR = 2.23, 95%CI: 1.43–3.47; *p* < 0.001) were independent risk factors for prediction.

**Table 3:** Results of multivariate logistic regression model.

Items	Multivariate logistic regression	
	OR (95% CI)	*p* value
Gender (male, %)	0.66 (0.47, 0.94)	0.020
BMI (kg/m^2^)	0.93 (0.89, 0.98)	0.004
STEMI (%)	2.23 (1.55, 3.20)	<0.001
HbA1c (%)	1.11 (1.03, 1.21)	0.010
LYMPH (10^9/L)	0.81 (0.63, 1.04)	0.103
PCT (ng/dL)	0.97 (0.92, 1.03)	0.387
TG (mmol/L)	0.90 (0.77, 1.06)	0.210
Lipoprotein (a) (mg/L)	1.00 (0.99, 1.00)	0.162
NT-ProBNP (pg/mL)	1.00 (1.00, 1.00)	0.348
CK (U/L)	1.00 (1.00, 1.00)	0.205
LVEF (%)	0.92 (0.91, 0.94)	<0.001
PH (%)	2.36 (1.36, 4.09)	0.002
Pericardial effusion (%)	2.23 (1.43, 3.47)	<0.001

OR, odds ratio; CI, confidence interval; other abbreviations as in Table 1.

**Figure 3:**
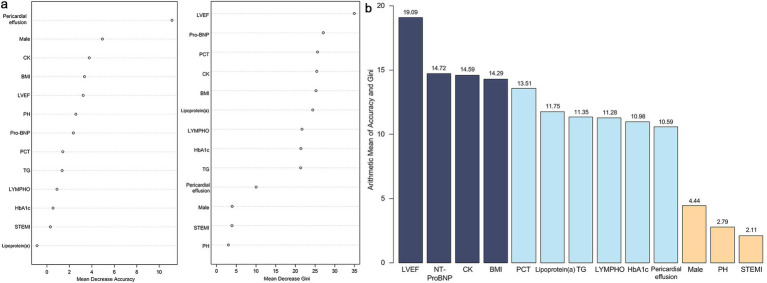
Results of random forest model. **(a)** Mean decrease accuracy and mean decrease Gini index of all variables. **(b)** Arithmetic mean value between mean decrease accuracy and mean decrease Gini index of all variables. LVEF, left ventricle ejection fraction; NT-ProBNP, N-terminal pro-brain natriuretic peptide; CK, creatine kinase; BMI, body mass index; PCT, procalcitonin; TG, triglycerides; LYMPH, lymphocyte count; HbA1c, hemoglobin A1c; PH, pulmonary hypertension; STEMI, ST-segment elevation myocardial infarction.

Furthermore, a random forest model analysis was performed following the LASSO-logistic regression (Figure 3). The mean accuracy and Gini index for each variable were calculated and subsequently ranked based on the arithmetic means of the accuracy and Gini index values. Given the numerical character of the arithmetic mean, the top four variables were included in subsequent analyses, which encompassed LVEF, NT-ProBNP, CK and BMI.

### 3.4 Construction of nomogram and its evaluation

**Figure 4:**
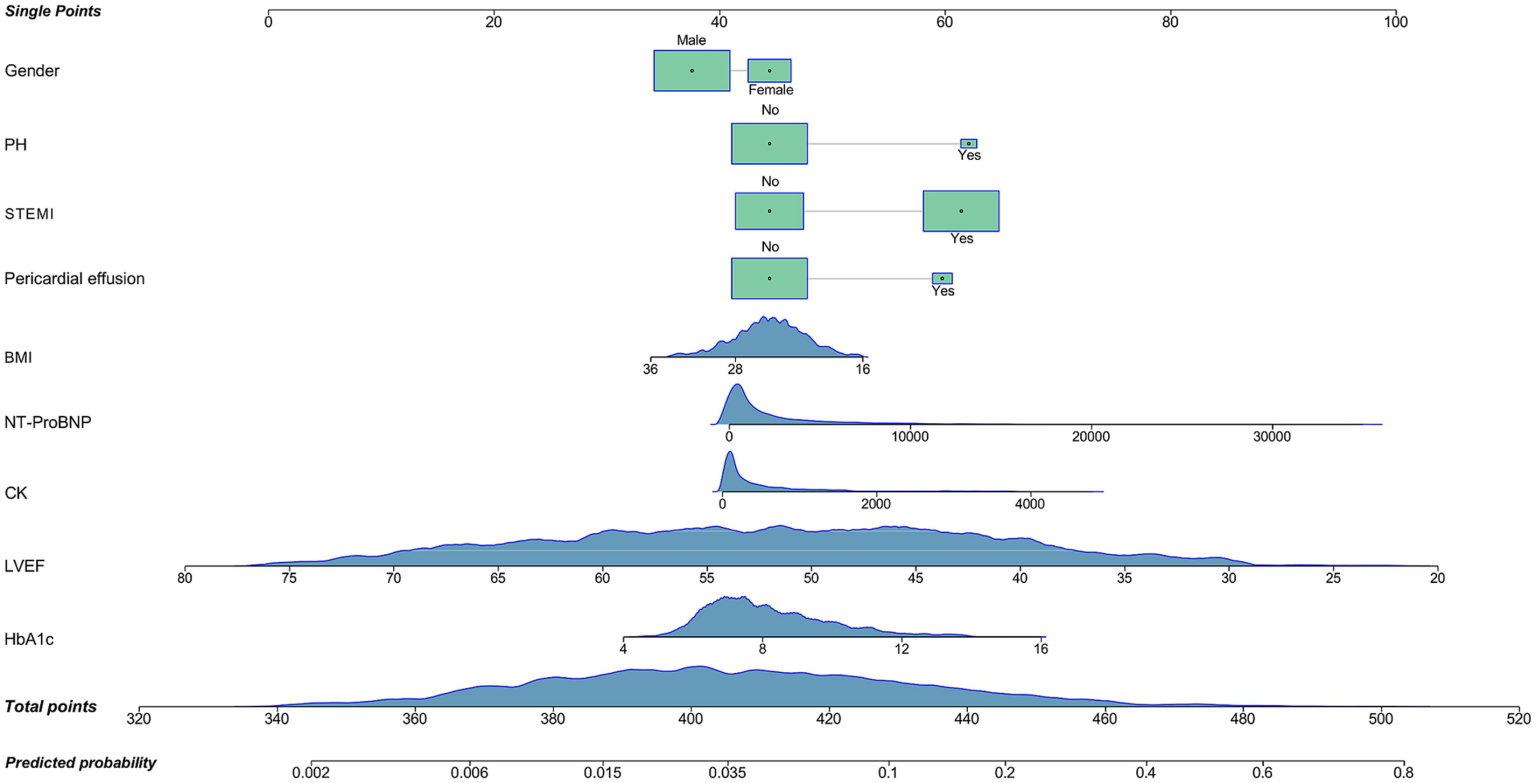
Nomogram for the prediction of mechanical complications in acute myocardial infarction patients with type 2 diabetes mellitus. PH, pulmonary hypertension; STEMI, ST-segment elevation myocardial infarction; BMI, body mass index; NT-ProBNP, N-terminal pro-brain natriuretic peptide (reference range for healthy populations: 0–125 ng/mL; diagnosis range for acute heart failure in people less than 50 years old: >450 ng/mL; diagnosis range for chronic heart failure: >2,000 ng/mL; upper detection limit: 35,000 pg/mL); CK, creatine kinase (reference range: 40–200 U/L); LVEF, left ventricle ejection fraction (reference range: 52–75%); HbA1c, hemoglobin A1c (reference range: 4.0–6.0%).

**Figure 5:**
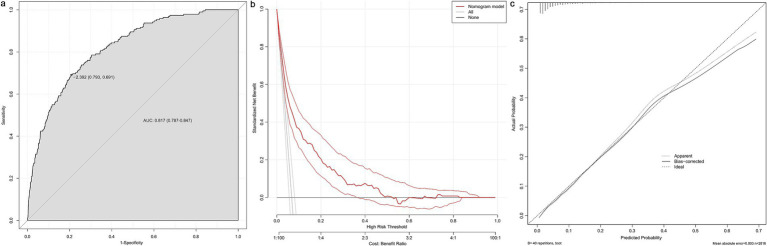
Evaluation of the nomogram. The receiver operating characteristic curve **(a)**, decision curve **(b)** and calibration curve **(c)** of the nomogram. AUC, area under curve.

Based on variable selection by multivariate logistic regression and random forest modeling, nine variables were used to construct a nomogram, including gender, pulmonary hypertension (PH), STEMI, pericardial effusion, BMI, NT-ProBNP, creatine kinase (CK), LVEF and HbA1c (Figure 4). For continuous variables, the shaded areas under the curve visually represent the distribution of individuals across different ranges, whereas for categorical variables, color blocks illustrate the relative proportions within each category. According to biochemical results and characteristics of patients, each variable was assigned specific points. The total score, calculated by summing all nine component scores, corresponded to the probability of experiencing in-hospital mechanical complications. The receiver operating characteristic curve indicated an area under the curve of 0.817 (95% CI: 0.787–0.847). Both the decision curve and calibration curve confirmed that the nomogram possessed good accuracy and consistency for clinical assessment (Figure 5).

## 4 Discussion

Although the reperfusion therapies have significantly improved outcomes for AMI patients, mechanical complications continue to pose challenges regarding both short-and long-term prognosis, particularly in patients with concurrent T2DM. This study focused on AMI patients with T2DM, dividing them into complication and control groups. Following the analysis of their baseline characteristics upon admission, nine risk factors for in-hospital mechanical complications were identified. The complication group also exhibited a higher proportion of in-hospital mortality and extracorporeal life support device usage, indicating a poorer prognosis for this population.

T2DM is increasingly prevalent among AMI patients, with an incidence rate of approximately 25% (11). This trend may be attributed to the prolonged hyperglycemia experienced in T2DM patients, which can lead to platelet and endothelial dysfunction, thereby accelerating the atherosclerosis of coronary arteries. From a treatment perspective, somehow, it has been observed that the receiving rates of coronary angiography and percutaneous coronary intervention are lower in T2DM patients compared to their non-diabetic counterparts, which may contribute to worse outcomes, including mechanical complications (12). Furthermore, T2DM patients demonstrate limited left ventricular remodeling following AMI, which also elevates the risk of complications. A study indicated that even patients with transient hyperglycemia are more likely to develop in hospital complications (11). However, this study found that the long-term blood glucose control could benefit patients even after the onset of AMI, and elevated HbA1c levels could potentially serve as a predictor for mechanical complications, highlighting the importance of intensive glycemic management. Notably, the use of metformin post-AMI is also associated with reduced mortality rates (13). Emerging evidence also suggests that in T2DM patients, early and intensive glycemic control, along with the use of glucose-lowering medications, can help reduce the future risk of major adverse cardiovascular events, including stroke, AMI, and all-cause mortality (14). The implementation of timely and rapidly blood glucose control within 24 h of hospital admission may be advantageous in reducing the risk of 30-day mortality (15). Additionally, the triglyceride glucose index, calculated from triglycerides and fasting blood glucose, may serve as a predictor for major adverse cardiac and cerebral events over a median follow-up period of approximately 2 years (16).

There are also several studies focusing on the prediction of mechanical complications. A study found that the neutrophil-to-lymphocyte ratio, as an inflammatory marker, serves as an independent predictor for the occurrence of mechanical complications and left ventricular dysfunction in patients with ST-elevation myocardial infarction (17). In this study, although the neutrophil-to-lymphocyte ratio was included in the analysis, it did not demonstrate a significant difference. It is speculated that the influence of blood glucose may be more pronounced than inflammation in AMI patients combined with T2DM, further underscoring the importance of glucose control. Another study indicated that AMI patients experiencing mechanical complications might have fewer comorbidities such as hypertension, diabetes, and dyslipidemia. However, mechanical complications are more likely to be associated with patients who do not have malignancies or a history of AMI (18). These results contrast with our findings, as our patient cohort exhibited higher levels of blood lipids. Meanwhile, one study indicated that females have higher risk of mechanical complications and associated mortality, as females are less likely to receive necessary operations compared to males, which is consistent with results of this study (19). Overall, further research is needed to better elucidate the risk factors associated with mechanical complications.

After the onset of mechanical complications, prompt surgical interventions combined with preoperative or postoperative extracorporeal life support devices are essential. These may include intra-aortic balloon pumps, extracorporeal membrane oxygenation, implantation of ventricular assist devices, and even heart transplantation, as shown in this study (7, 20). However, due to limited ECMO cases (*n* = 6) in this study, its impact on survival could not be reliably quantified, warranting further investigation. It has been suggested that achieving hemodynamic stabilization prior to surgery may enhance survival rates postoperatively, particularly in patients with ventricular septal defects (20). However, despite receiving necessary therapies, the in-hospital mortality rate for AMI patients with mechanical complications remains around 50%, especially among older individuals, those with heart failure, cardiogenic shock or renal dysfunction (6, 21, 22). Furthermore, the literature assessing outcomes of surgical interventions presents considerable differences. Among all mechanical complications, ventricular septal defect repair appears to yield the most favorable results (23). Some studies have indicated that long-term survival rates for patients undergoing surgical procedures are promising. The 5-year and 10-year survival rates are approximately 48.1 and 41.0%, respectively (24). However, another study indicated that concomitant coronary artery bypass grafting may not necessarily result in lower early or late mortality, rendering the benefits of revascularization controversial (25). Meanwhile, a study found that the extracorporeal life support devices may only improve the clinical outcomes in patients with pseudoaneurysm and papillary muscle rupture, but do not significantly affect the overall survival (18). Overall, the management of AMI patients with mechanical complications requires further improvement, which includes the selection of appropriate life support devices and determination of optimal timing for surgical intervention.

This study also has certain limitations. Firstly, the conditions experienced during hospitalization were not included in the analysis, which may also influence the occurrence of mechanical complications. Furthermore, as this is a retrospective study, some relevant factors may be unable to obtain. Additionally, prospective analyses with long-term follow-up are necessary to more comprehensively evaluate the effectiveness of the nomogram.

## 5 Conclusion

This study found that gender, PH, STEMI, pericardial effusion, BMI, NT-ProBNP, CK, LVEF and HbA1c upon admission were associated with in-hospital mechanical complications among AMI patients who have a history of T2DM. A nomogram was constructed based on these risk factors to facilitate earlier identification and intervention of high-risk patients. It is imperative for individuals with T2DM to manage their blood glucose levels effectively since this may provide advantages even after experiencing an episode of AMI.

## Data Availability

The raw data supporting the conclusions of this article will be made available by the authors, without undue reservation.
